# High quality diet improves lipid metabolic profile and breeding performance in the blue-footed booby, a long-lived seabird

**DOI:** 10.1371/journal.pone.0193136

**Published:** 2018-02-20

**Authors:** Erick González-Medina, José Alfredo Castillo-Guerrero, Sharon Zinah Herzka, Guillermo Fernández

**Affiliations:** 1 Posgrado de Ciencias del Mar y Limnología, Universidad Nacional Autónoma de México, Ciudad Universitaria, México D.F., Mexico; 2 CONACYT- –Universidad de Guadalajara, Departamento de Estudios para el Desarrollo Sustentable de la Zona Costera, Centro Universitario de la Costa Sur, San Patricio– Melaque, Municipio de Cihuatlán, Jalisco, Mexico; 3 Departamento de Oceanografía Biológica, Centro de Investigación Científica y de Educación Superior de Ensenada (CICESE), Baja California, Mexico; 4 Unidad Académica Mazatlán, Instituto de Ciencias del Mar y Limnología, Universidad Nacional Autónoma de México, Mazatlán, Mexico; University of Lleida, SPAIN

## Abstract

Understanding the role of diet in the physiological condition of adults during reproduction and hence its effect on reproductive performance is fundamental to understand reproductive strategies in long-lived animals. In birds, little is known about the influence of the quality of food consumed at the beginning of the reproductive period and its short-term effects on reproductive performance. To assess the role of diet in the physiological condition of female blue-footed booby, *Sula nebouxii* (BFBO), during reproduction we evaluated whether individual differences in diet (assessed by using δ^13^C and δ^15^N values of whole blood from female birds and muscle tissue of the principal prey species) prior to egg laying and during incubation influenced their lipid metabolic profile (measured as triglyceride levels and C:N ratio) and their reproductive performance (defined by laying date, clutch size and hatching success). Females with higher δ^15^N values in their blood during the courtship and incubation periods had a higher lipid metabolic profile, earlier laying date, greater clutch size (2–3 eggs) and higher hatching success. Females that laid earlier and more eggs (2–3 eggs) consumed more Pacific anchoveta (*Cetengraulis mysticetus*) and Pacific thread herring (*Opisthonema libertate*) than did other females. These two prey species also had high amounts of lipids (C:N ratio) and caloric content (Kcal/g fresh weight). The quality of food consumed by females at the beginning of reproduction affected their physiological condition, as well as their short-term reproductive performance. Our work emphasizes the importance of determining the influence of food quality during reproduction to understand the reproductive decisions and consequences in long-lived animals.

## Introduction

Food availability and selection has a substantial effect on energetic and fitness costs and performance during reproduction and therefore influences how individuals adjust their reproductive strategies [1,2,3]. Thus, it is not surprising that the nutritional quality of the available food influences the reproductive success and decisions in natural populations of many animals [4,5,6]. Despite extensive studies regarding the effect of food availability on maternal reproductive traits for specific taxa [5,6,7,8,9], the manner in which female reproduction responds to food availability and quality might differ among species and deserves further investigation [10].

Food availability/quality and age of the breeding birds can significantly affect female reproductive strategies [11,12,13,14,15]. In long-lived species, the quality of a female’s diet before egg formation and incubation can influence body condition, egg quality and reproductive parameters such as egg number, laying date, clutch size and breeding success [16,17,18]. Females with better body condition usually nest earlier in the breeding season and have a larger clutch size and greater reproductive success than those in poorer condition [19,20]. Hence, the nutritional quality and specific prey species consumed before the chick-rearing stage can affect the body condition and subsequent reproductive performance.

Several studies have evaluated the effect of diet on reproductive performance. The food consumed has been linked (by measuring the nitrogen stable isotope composition—δ^15^N values- of feathers as indicators of dietary trophic level) with laying date and egg volume in cassin's auklet (*Ptychoramphus aleuticus*) [8]. Females feeding on high-quality prey with a higher energy content (Kcal/g) during the pre-breeding period were able to breed earlier and produce larger eggs. However, the body condition of females was unrelated to the quality of the diet or the laying date [8]. The marbled murrelet (*Brachyramphus marmoratus*) had a positive relationship between the energy content (correlated with trophic level) of pre-breeding diet and reproductive success; in years when females were feeding on lower-quality prey during the pre-breeding period, fledging success was lower [21]. These studies indicate there is evidence relating some components of breeding performance to diet, but the proximal mechanisms are mostly undescribed, and the information available does not allow for discerning a clear pattern in seabirds. Moreover, under variable environmental regimes in tropical seabirds could promote different strategies to allocate resources to breeding.

Plasma metabolite concentrations reflect the physiological state of a bird in relation to fattening or fasting [22,23,24]. Plasma concentrations of metabolites related to lipid deposition, such as triglyceride, increase during feeding, fat accumulation [25], and where high-quality food are abundant [26]. Also, they are a good indicator of short-term changes in body reserves [25,27]. In female birds, triglycerides are related to the presence of egg precursors in blood [28] and to fat metabolism, with higher triglyceride levels in the blood considered indicative of better nutritional status [29]. Some studies have reported higher triglyceride levels in breeding females than in non-breeding females [28,30]. Before incubation, seabirds need to store enough nutritional reserves to endure the incubation period during which they spend long periods fasting. In birds, the interpretation of triglyceride levels in some studies have been complicated by processes such as the effect of diet quality [31,32]. Diet composition can affect plasma metabolite profiles independently of differences in rates of mass change, which could complicate the interpretation of plasma triglyceride concentrations as indicators of physiological state [31]. If there is a correlation between δ^15^N values and triglyceride levels during the fasting state, however, it might help the understanding of the role of the quality of the diet consumed and short-term consequences on the physiological condition of individuals and their reproductive performance.

Dietary lipids are energy-dense and are therefore likely to enhance reproductive performance in birds [33]. The C:N ratio in a tissue sample can be used as a proxy for the relative amount of lipids in aquatic animals such as fish [34] owing to the large amounts of carbon in lipids. Estimating caloric intake (Kcal/g) through the characterization of the prey consumed can provide information regarding the energy value and quality of specific prey types in relation to reproductive performance. Fish species that occupy a higher trophic level can have greater *per capita* caloric value than those from a lower level [35] and may have a higher lipid content as well [33]. Hence, foraging on higher trophic levels can increase reproductive performance through the increased provision of metabolic energy [36].

Stable isotope analyses are increasingly used in studies of marine predators and studies of seabirds have used them as proxies of diet quality [3,8,37]. δ^13^C values show a higher enrichment in inshore as compared to offshore food webs [38] and also reflect differences between planktonic and benthic primary production [39]. δ^15^N values increase with each successive trophic level and are therefore frequently used to estimate the trophic level of consumers [40]. Stable isotope values of whole blood samples are useful for assessing how the diet of individuals relates to their body condition because it integrates the diet assimilated over the previous 3–4 weeks [41].

The present paper concerns the blue-footed booby (*Sula nebouxii*, BFBO), in which, females can lay 1–3 eggs (most frequent two) [42]. The species feeds on small pelagic fish such as anchovies and sardines [43] that adults capture by plunge-diving [42]. In our study colony, on Isla El Rancho, Sinaloa, 16 prey species have been identified. The most common prey during 2003 and 2004 were Pacific thread herring (*Ophistonema libertate*) and Pacific anchoveta (*Cetengraulis mysticetus*) [43]. Females show a broad variation in laying date and other reproductive parameters, as well as in their body condition. For example, the difference between early and late breeders is approximately 90 days on Isla El Rancho, Sinaloa (Gonzalez-Medina E. unpublished data) and up to 161 days on Isla Isabel, Nayarit [44]. At the population level, annual food shortages associated with the El Niño Southern Oscillation (ENSO) can delay the onset of reproduction, with a consequent negative effect on other reproductive parameters, such as clutch size, brood size, hatching and fledging success [45]. It, therefore, seems likely that variations in food availability and quality can affect the body condition and reproductive performance of individual females within and among breeding seasons.

In this study we examined whether differences in the diet (prey composition as evaluated through the use of isotope mixing models, trophic level of the prey and C:N ratios as a measure of lipid levels) of individual BFBO females before the chick-rearing period influenced their lipid metabolic profile (based on triglyceride levels and C:N ratio) and reproductive performance. We predicted that females that feed at higher trophic levels, on prey with higher energy (Kcal/g) and with higher lipid content (C:N ratio) before the chick-rearing period (courtship and incubation) would present a more positive lipid metabolic profile (measured as higher triglyceride levels and C:N ratio) and better reproductive performance (earlier breeding, more eggs and greater hatching success).

## Materials and methods

### Ethics statement

The work met the Mexican legal requirements about animal welfare, and field work was annually supervised and approved by Dirección General de Vida Silvestre, Secretaría de Gestión para la Protección Ambiental (SGPA/DGVS/08559/11, SGPA/DGVS/62712/12).

### Study area and data collection

Fieldwork was conducted at Isla El Rancho (25°10’N, 108°23’W; 327 ha), in the northern mouth of Bahía Santa María-La Reforma, which is the largest coastal wetland of Sinaloa, Mexico [46]. About 3,000 pairs of BFBO nest in the island. We visited the island between December and May during two breeding seasons (2011 and 2012). We randomly selected a series of nests to be monitored throughout each breeding season. The courtship period (the period after pairing, previous to the production of eggs, when mates display a set of behaviors associated with sexual union, lasts a relatively long time (up to 40 days) [47], was evaluated only during the 2012 season (22 pairs monitored), whereas the incubation period was evaluated during both seasons (18 pairs monitored in 2011 and 14 pairs in 2012, of which 13 were repeated from the courtship period). We recorded laying date, clutch size, egg volume per clutch and hatching success (eggs hatched / total eggs laid ×100). When the exact laying date was not recorded, it was estimated based on the date of the first hatching chick and subtracting 42 days of incubation. The value used as laying date was the number of days after the first nest was observed at the beginning of the breeding season. During the breeding season (2011–2012), the modal laying date was 7 December (28 days after the first egg laid, *n* = 154; range 10 November—14 February). We used lay date to classify early and late breeding birds, so nests that were found prior to the modal date of the entire breeding season were classified as early breeders and those that were above were classified as late breeders. Eggs were measured with callipers (±0.1 mm) and egg volume (cm^3^) was calculated as maximum length × maximum width^2^ × 0.51/1000 [48], a method previously used for this species [49]. The total egg volume per clutch (hereafter ‘clutch size’) was calculated by adding the individual egg volumes per clutch.

### Sampling blue-footed booby and prey

Females were individually marked with alphanumeric bands. We caught females in their nests, collected a sample (about 1.5 ml) of whole blood from the brachial vein and transferred this to two polypropylene tubes (0.5 ml and 1 ml respectively) without anticoagulant. The true plasma triglyceride level (calculated by subtracting glycerol from total triglyceride) was obtained by centrifugation, while for stable isotopes analysis (δ^13^C and δ^15^N) whole blood was used. We obtained the C:N ratio (as an indicator of lipid content) from the blood. The time between capture and blood collection was always < 15 min (9.6 ± 3.5 min; mean ± SD). All samples were kept on ice in the field and frozen in the laboratory pending preparation for their respective analysis. In seabirds, the isotopic composition of whole blood reflects the diet about 30 days earlier [40]. The difference in days between samples from birds caught at the 13 nests that were sampled during both courtship and incubation periods were 32.3 ± 7.9 days. Samples collected from the other nests correspond to either the courtship or the incubation period.

We collected reference samples of the most common fish prey species of the BFBO for isotope analysis. Prey that was regurgitated when we handled the birds or regurgitated spontaneously when we walked through the colony were collected only if the specimens were complete and exhibited minimal digestion (Table 1). We collected at least six individuals per fish prey species. These samples were kept on ice in the field and were frozen in the laboratory. Prey species were identified in the field and in the laboratory. A small portion of dorsal muscle from each prey was dissected for stable isotope analyses. We obtained the C:N ratio from the muscle of the principal fish prey; also, we obtained the caloric intake (Kcal/g fresh weight) of each primary prey from literature.

**Table 1:** δ^13^C and δ^15^N values (average +/- SD), C:N ratio (as an indicator of lipid content) and estimated energy content (Kcal/g fresh weight) of the principal prey species of blue-footed boobies (*Sula nebouxii*). Group 1 (*Scomber australasicus*, *Engraulis mordax*, and *Decapterus macarellus*).

Prey species	n	δ^13^C (‰)	δ^15^N (‰)	C:N ratio	Kcal/g (fresh weight)
*Anchoa* spp	29	-16.59 ± 0.6	17.45 ± 0.7	3.4	5.30^a^
*Cetengraulis mysticetus*	37	-15.85 ± 1.3	17.47 ± 0.7	5.6	6.96^b^
*Opisthonema libertate*	21	-15.16 ± 1.6	17.85 ± 0.4	5.9	4.29^b^
*Hyporhamphus unifasciatus*	6	-16.44 ± 0.6	17.89 ± 1.4	3.3	1.41^c^
*Hemiramphus saltator*	7	-17.2 ± 1.0	18.49 ± 0.9	3.5	1.51^c^
Group 1	21	-17.47 ± 0.9	17.59 ± 0.7	3.8	1.56^d^

^a^[50]

^b^[51]

^c^[52]

^d^[52,53,54]

### Sample preparation and stable isotope analysis

Before isotope analysis, whole avian blood and fish muscle were dried in an oven at 60°C for approximately 24 h and then ground into fine powder. Dried samples were weighed and wrapped in tin capsules. Weights ranged from 700 to 1000 mg. Samples were sent to the Stable Isotope Facility (SFU) at the University of California, Davis, USA for analysis of δ ^13^C and δ^15^N. Isotope ratios are reported using standard notation: *δ*_*sample*_ = ((*R*_*sample*_ − *R*_*standard*_) / *R*_*standard*_)* 1000 where δ_*sample*_ is the isotopic ratio of the sample and R is the ratio of the heavy to light isotope (^13^C/^12^C or ^15^N/^14^N) in the sample relative to V-PDB or atmospheric nitrogen, respectively. Secondary standards [mean (SD)] analyzed during the sample runs were bovine liver [δ^13^C = −21.69‰ (SD 0.03‰), δ^15^N = 7.72‰ (SD 0.17‰)], USGS-41 glutamic acid [δ^13^C = 37.62‰ (SD 0.06‰), δ^15^N = 47.6‰ (SD 0.21‰)], Nylon 5 [δ^13^C = −27.72‰ (SD 0.04‰), δ^15^N = −10.31‰ (SD 0.15‰)] and glutamic acid [δ^13^C = −28.85‰ (SD 0.03‰), δ^15^N = −4.26‰ (SD 0.23‰)]. Hence, the precision of isotope measurements was < 0.24‰ for both nitrogen and carbon stable isotopes.

### Total triglyceride analysis

Plasma levels of total triglycerides (triglyceride plus free glycerol) were assayed in a multiparameter chemistry analyzer (Falcor 360; Menarini Diagnostics, Barcelona, Spain) with commercial kits (Menagent; Menarini Diagnostics) adapted for small sample volumes [55]. The analyzer was calibrated with a commercial calibrator kit (Menagent; Menarini Diagnostics) and control reference serums (Menagent; Menarini Diagnostics) were run together with plasma samples. Plasma levels of glycerol were assayed by means of standard diagnostic kits in a microplate spectrophotometer (BioTek Powerwave, Winooski, VT, USA) using 400-μl flat-bottom microplates (Greiner Bio-One, Frickenhausen, Germany) by endpoint assay with the commercial kits adapted for small-volume samples as described by [36]. Specifically, we used free glycerol reagent (Sigma, Stockholm, Sweden, 2.5 μl plasma, 200 μl reagent). All measurements were made in duplicate. Because the triglyceride assay also measures free glycerol, true plasma triglyceride levels were calculated by subtracting glycerol from total triglyceride.

### Statistical analyses and isotope mixing models

We used a simple linear regression to evaluate the relationship between the trophic level (inferred from δ^15^N values), and lipid metabolic profile (triglyceride levels and C:N ratio as an indicator of lipid content) of BFBO females during courtship, with triglyceride levels or C:N ratio as a response variable, and δ^15^N values as an independent variable. To evaluate the relationship between the trophic level, and lipid metabolic profile of females during incubation we used a general linear model (LM), with triglyceride levels or C:N ratio as a response variable, year (2011 or 2012) as a categorical variable and δ^15^N values as a continuous variable. To assess whether the isotope composition (δ^15^N) of whole blood of females during the courtship period was related to reproductive parameters, we used an analysis of simple linear regression for each variable, with laying date and clutch size as response variables and δ^15^N values as a continuous independent variable. To determine the relationship between the isotopic composition (δ^15^N) in whole blood during the incubation period and hatching success, we ran a generalized linear model (GLM) with a binomial distribution and logit link function (successful hatching = 1, no hatching = 0); year (2011 and 2012) and laying order (1–3) were considered as a categorical variables and δ^15^N values as a continuous variable. The Wald test was used to test the statistical significance of each coefficient in the model. Statistical analysis used Statistica 10 [56]. The values reported are the mean ± SE. Significance levels were set at 0.05.

We used the δ^13^C and δ^15^N values of whole blood from female birds and muscle tissue of the principal prey species to estimate the fractional contribution of potential prey to the diets of nesting females (divided into early and late breeders) and as a function of clutch size (0–3 eggs). We used the SIAR Bayesian multi-source isotopic mixing model in the R environment [57] to quantify the proportion of each prey in the diet of females. The SIAR model can be used to estimate source (prey) contributions to consumer tissues while accounting for the observed variability in source and mixture isotopic ratios, dietary isotopic fractionation and elemental concentration by using a Bayesian approach to estimate the probability distributions of multiple-source [58]. To decrease the number of sources, species that did not differ in isotopic composition (δ^13^C and δ^15^N), Californian anchovy (*Engraulis mordax*), Blue mackerel (*Scomber australasicus*) and Mackerel scad (*Decapterus macarellus*) were grouped as “Group 1”. Isotopic values were pooled prior to incorporation into the SIAR mixing model.

As there have been no controlled studies to determine boobies-specific trophic enrichment factors, we used whole-blood isotope discrimination factors derived from captive controlled studies of a piscivorous bird to correct for tissue-diet trophic isotope fractionation (see review by [59]; δ^13^C: 0.24 ± 0.79‰; δ^15^N: 2.25 ± 0.61‰), these values have already been used in other study with northern gannets *Morus bassanus* a closely related species with the BFBO [60].

## Results

### Trophic level and lipid metabolic profile

**Fig 1:**
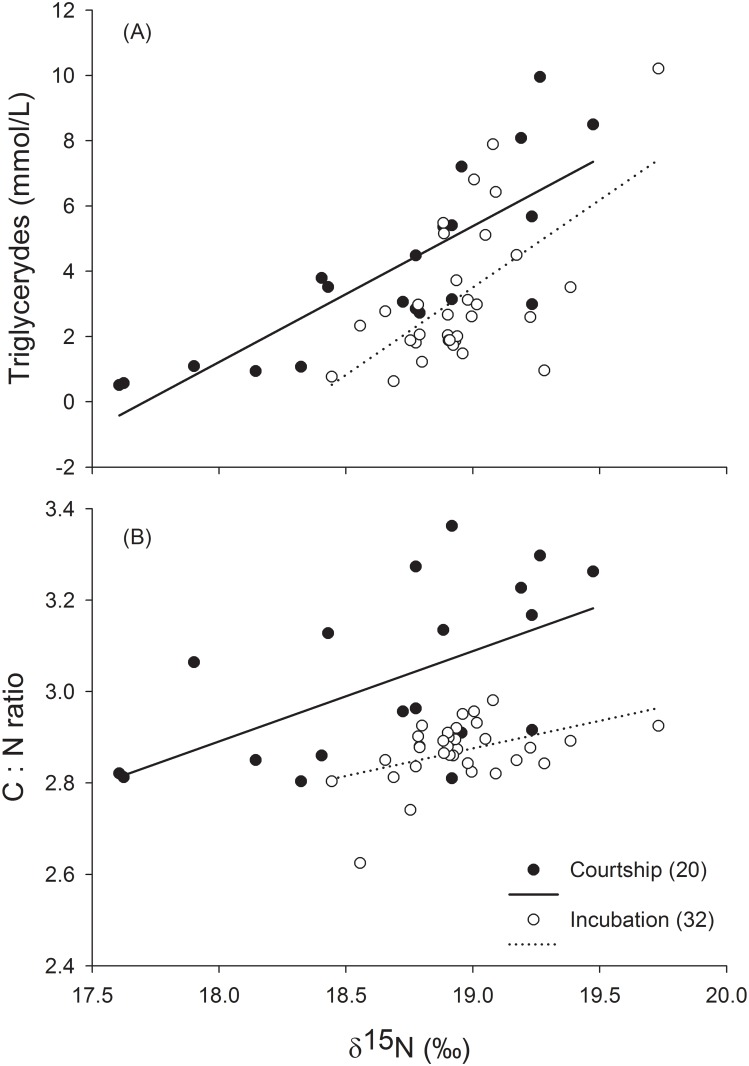
Relationship between δ^15^N values measured in female blue-footed booby (*Sula nebouxii*) whole blood during two reproductive periods (courtship and incubation, sample sizes in parentheses) and (A) triglyceride levels (indicator of body condition) and (B) C:N ratio.

The lipid metabolic profile of females (as measured by triglyceride levels and C:N ratio) were positively related to δ^15^N values during courtship (triglyceride: *F*_*1*,*18*_ = 34.74, *r*^2^ = 0.66, *β* = 4.16, *P* < 0.001; C:N ratio: *F*_*1*,*18*_ = 8.26, *r*^2^ = 0.31, *β* = 1.6, *P <* 0.05) and incubation (triglyceride: *F*_*1*,*29*_ = 20.44, *r*^2^ = 0.41, *β* = 0.66, *P* < 0.001; C:N ratio: *F*_*1*,*30*_ = 7.17, *r*^*2*^ = 0.16, *β* = 1.59, *P <* 0.05; Fig 1). Breeding season had no effect on lipid metabolic profile during the incubation period (triglyceride: *F*_*1*,*29*_ = 3.62, *P* = 0.07; C:N ratio: *F*_*1*,*29*_ = 0.59, *P* = 0.44).

### Diet quality and reproductive variables

**Fig 2:**
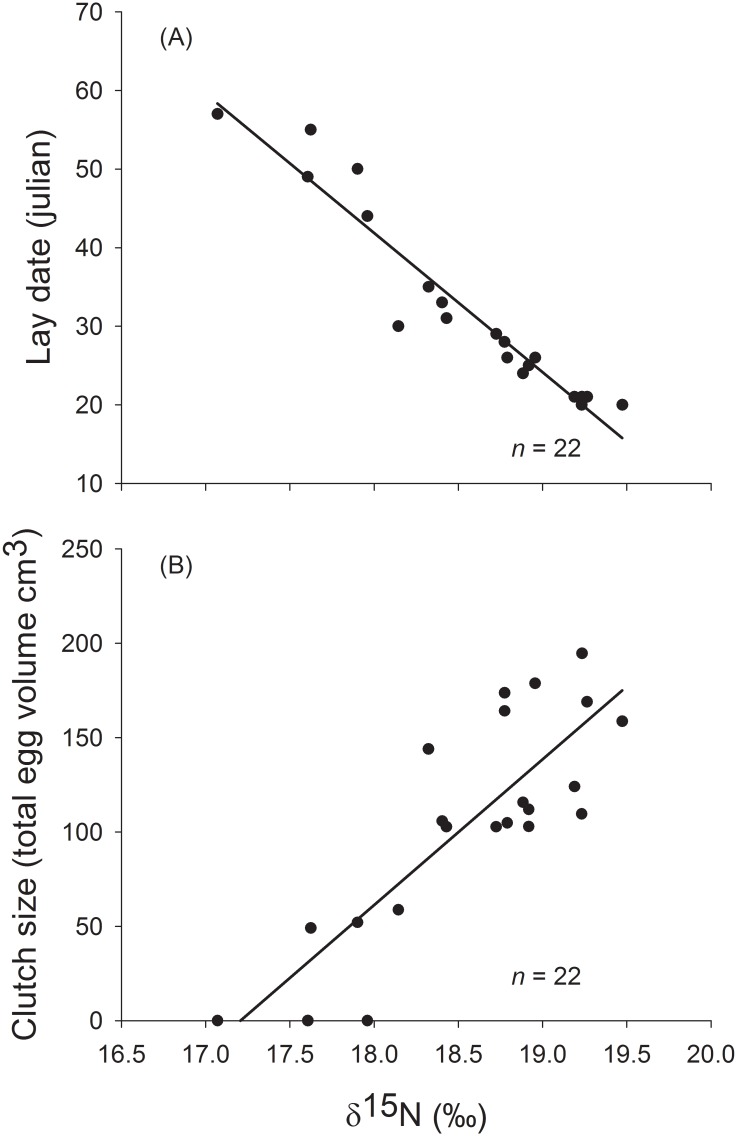
Relationship between δ^15^N values measured in female blue-footed booby (*Sula nebouxii*) whole blood during the pre-laying period and (A) lay date and (B) clutch size (total egg volume from the clutch).

**Fig 3:**
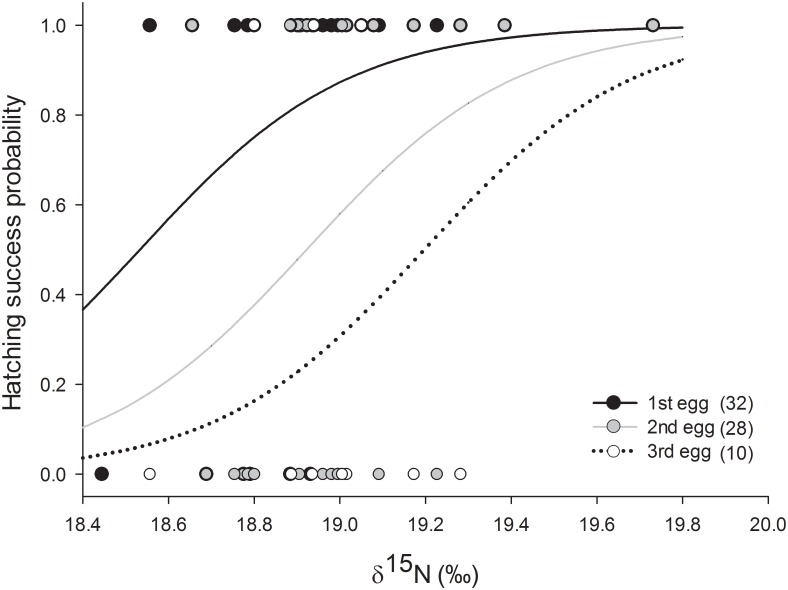
Relationship between δ^15^N values measured in female blue-footed booby (*Sula nebouxii*) whole blood during the incubation period and hatching success (sample sizes in parentheses, data and estimated probability). Fitted lines (black, grey and dotted) are derived in GLM models and represents the laying order (1–3).

During courtship, there was a negative relationship between δ^15^N values and laying date (*F*_*1*,*20*_ = 239.24, *r*^*2*^ = 0.92, *β* = -17.71, *P* < 0.001) and a positive relationship between δ^15^N values and clutch size (*F*_*1*,*20*_ = 47.73, *r*^*2*^ = 0.70, *β* = 77.2, *P* < 0.001), also there was a positive relationship between δ^15^N values and hatching success (*Wald* = 5.99, *df* = 1, *P* = 0.01). There were differences in hatching success related to laying order (*Wald* = 10.3, *df* = 2, *P* = 0.01) but not between the two breeding seasons (*Wald* = 0.08, *df* = 1, *P* = 0.77). Hence, high δ^15^N values were related to early laying date, increased clutch size and better hatching success (Figs 2A, 2B and 3). Moreover, 2nd and 3rd eggs had a progressive diminish of hatching success (Fig 3).

### Prey composition

We collected 121 individual fish representing eight prey species (Table 1) during two breeding seasons, except Mackerel scad and Californian anchovy, which were collected only during 2011 and Common halfbeak (*Hyporhamphus unifasciatus*), which was collected only in 2012. For those species that were sampled during both breeding seasons, the isotopic composition did not differ between seasons (*P* > 0.05), and so the data were pooled and applying mixing models. C:N ratios in fish muscle were highest in Pacific anchoveta and Pacific thread herring and were lowest in Common halfbeak and anchovy (*Anchoa* spp) (Table 1). The energy values of the primary prey species ranged from 6.96 to 1.41 Kcal/g fresh weight (Pacific anchoveta and Common halfbeak, respectively; Table 1). Mean carbon and nitrogen isotope ratios of potential prey exhibited a limited range of values (−15.2—−17.5‰ and 17.4–18.5‰, respectively).

Based on the isotopic mixing models, our results show that the diet composition of female birds had small variations between early and late breeders (Table 2) and varied as a function of clutch size (Table 2). Apparently, females consumed more Pacific anchoveta and Pacific thread herring, which accounted for ≈ 56–50% of total consumption (Table 2). These two prey species likely had the highest amount of lipids in their blood (indicated by a high C:N ratio) and had high caloric content (Kcal/g fresh weight). Females nesting later were estimated to have a slight decrease in consumption of Pacific anchoveta and Pacific thread herring and a slight increase in consumption of Longfin halfbeak and Group 1, but estimated ranges largely overlap (Table 2). Females with a clutch size of 2–3 eggs consumed mainly Pacific anchoveta and Pacific thread herring, which accounted for ≈ 69–54% of total consumption respectively (Table 2). Females with a clutch size of 0–1 eggs consumed relatively even proportions of all prey species (< 21% / species) (Table 2).

**Table 2:** Fractional contribution of prey of female blue-footed booby (*Sula nebouxii*) estimated based on δ^13^C, and δ^15^N values of bird whole blood samples and regurgitated fish (*n* = 121) collected during two years (2011 and 2012). Group 1 (*Scomber australasicus*, *Engraulis mordax*, and *Decapterus macarellus*).

Prey species	Early breeders	Late breeders	0-eggs	1-eggs	2-eggs	3-eggs
*Anchoa* spp	15.68 (0–34)	16 (0–34)	16.91 (0–33)	17.11 (0–34)	12.44 (0–30)	14.96 (0–33)
*Cetengraulis mysticetus*	26.28 (1–49)	22.72 (0–43)	18.69 (0–36)	20.04 (0–38)	32.24 (4–59)	23.73 (0–45)
*Opisthonema libertate*	30.55 (8–52)	26.86 (2–51)	19.21 (0–37)	20.85 (0–39)	37.44 (15–59)	30.77 (3–59)
*Hyporhamphus unifasciatus*	13.22 (0–31)	14.65 (0–32)	16.46 (0–33)	16.25 (0–33)	9.24 (0–24)	13.55 (0–31)
*Hemiramphus saltator*	6.27 (0–18)	8.93 (0–24)	13.71 (0–30)	11.94 (0–28)	3.62 (0–10)	7.74 (0–22)
Group 1	8 (0–22)	10.83 (0–27)	15.02 (0–31)	13.8 (0–30)	5.02 (0–14)	9.25 (0–25)

Multi-source Bayesian isotopic mixing model SIAR [61] were applied by separating samples as a function of laying date (early and late breeders) and considering clutch size (0–3 eggs). Mean estimates of the fractional contribution (estimated values are % of prey consumption) with 95% credibility intervals (in parentheses) are reported.

## Discussion

Higher δ^15^N values in the blood of females during courtship and incubation were related to higher lipid metabolic profile (based on triglyceride levels and C:N ratio), earlier laying date, greater clutch size (2–3 eggs) and higher hatching success. Hence, the quality of the diet consumed by females of BFBO during the early stages of reproduction had short-term consequences for their physiological condition and reproductive performance.

Females nesting early need to obtain nutrients required for egg production [62]. Lipids and proteins (mainly albumin) are two of the main resources needed for egg formation [63] and their consumption and metabolism can affect the isotopic composition of the blood of birds [34,64]. For example, if endogenous protein stores are used as an energy substrate, the relatively higher δ^15^N values observed in blood samples could be the result of the consequent retention of the heavy isotope (^15^N) and excretion of light nitrogen. The lipid component of any given tissue type can be quite variable among individuals and generally is depleted in ^13^C compared with whole tissues [65]. Females of BFBO have higher triglyceride levels prior to egg laying, whereas butyrate (indicator of the use of body reserves) and albumin levels in blood serum show no variations during breeding [66]. High triglyceride levels indicate that there is lipid as an energy source and therefore it is likely that high δ^15^N values are not a reflection of protein catabolism; rather, it is due to selectivity in the diet of females. The positive relationship between lipid metabolic profile (triglyceride levels and C:N ratio) and δ^15^N values during courtship and incubation is consistent with this explanation. Likewise, this particular result might help yield insight into the interpretation of triglyceride levels in birds as indicators of physiological state, given that food quality (as measured through δ^15^N values and caloric and lipid content) was positively correlated with triglyceride levels, but should be interpreted with caution, as other potential confounding factors (e.g., recent food ingestion, physiological stress) were not measured. This is a new approach that can contribute toward the understanding of what plasma triglycerides measure or correlate with in seabirds and whether or not they serve as indicators of lipid metabolic profile. Another possible cause of enrichment in ^15^N in female blood could be seasonal variations of fish prey species, although more studies are needed to corroborate this.

Our results show a strong link between prey δ^15^N values (and hence trophic levels), food quality and lipid metabolic profile (as indicated by triglyceride levels and C:N ratio) and measures of reproductive performance. Females that had a higher lipid metabolic profile and reproductive performance fed mainly on Pacific anchoveta and Pacific thread herring, both were prey species with a high content of available energy (Kcal/g fresh weight) and lipid content (as indicated by C:N ratios). Birds with a high rate of body mass gain have been shown to have high plasma triglyceride levels [67]. Some studies of seabirds have shown a significant positive effect on body condition (measured as body mass) associated with the consumption of prey enriched in ^15^N, including research on magellanic penguin (*Spheniscus magellanicus*) [68], thick-billed murre (*Uria lomvia*) and black-legged kittiwake (*Rissa tridactyla*) [69], whereas others have shown a significant negative relationship [70], or were unable to establish a significant relationship [8]. These apparently contradictory results may be due to the method of estimating body condition used in each study; all of these studies used body mass (corrected for size) as a measure of body condition, which may be an inadequate estimate, especially if the relationship is nonlinear [71]. Also, body mass provides only a static assessment of an individual’s performance at the time of capture [23]. Our results highlight the importance of short-term consumption of food in the physiology of females and then in the preferential allocation to breeding.

Individual variation in laying date has been associated with body condition, age and/or reproductive experience of the females [19,72]. Individuals with better body condition usually nest early during the breeding season, have a larger clutch size and show better reproductive performance than individuals of lower body condition [19,20]. Females that can quickly accumulate the reserves of body fat necessary for reproduction may differ in foraging efficiency [19]. Our results indicated that females with higher lipid metabolic profile that fed on high-quality prey species (and caloric and lipid contents), tended to breed early with a greater clutch size and had a higher hatching success than did females fed on low-quality prey species. This is consistent with previous suggestions that breeding success declines with laying date [20,73,74]. Our results are indicative of a mechanistic explanation for the decline in breeding performance with laying date.

Egg volume reflects the ability of females to assign nutrient reserves to egg formation [75] and is a trait with high plasticity driven by the feeding conditions prior to egg laying [76]. Egg formation requires nutrients, especially lipids and proteins [63]. In the case of BFBO females, the normal clutch size of 2 eggs represents approximately 7.2% of their mass [42]. Our results, like other studies [77,78], showed a positive correlation between clutch size and δ^15^N values (reflecting the trophic level at which feeding occurred). Females that had not nested or had laid a single egg presented lower δ^15^N values and lipid metabolic profile and the results of the mixing model and C:N ratios indicated they had fed on lower-quality prey species. The fact that some females did not nest and/or laid a single egg, may suggest a physiological limitation that prevents egg formation, thereby restricting reproductive investment. A disproportionate number of females with smaller clutch size may be young adults that are breeding for the first time with less experience in foraging. Younger birds have been shown to have a lower body mass at the beginning of the breeding season and lower success with prey capture [79], which could reduce their ability to assign reserves to egg production and breeding.

Body condition has been widely used as a predictor of reproductive success in birds and it has also been postulated that the amount of reserves at the beginning of reproduction, regulated by environmental conditions, influences the expenditure of energy on reproduction [80]. This implies that the quality of food consumed by females may directly affect their reproductive performance in the short term. There are some studies linking body condition of females with reproductive success [16,81]. When the birds are facing food shortages at the beginning of the reproductive period, females can adapt in the short term by reducing the clutch size [82], or the size of the eggs [83]. In this context, the quantity and/or quality of food consumed is of great importance. The junk-food hypothesis postulates that seabirds that feed on low-quality prey (with little nutritional value and energy) exhibit reduced reproductive success [84] because it adversely affects the growth patterns, body condition and cognitive abilities of their offspring [85]. However, according to our results, females with higher triglyceride levels that fed on high-quality prey species with high caloric and lipid contents tended to breed early with a greater clutch size and had a higher hatching success than did females fed on low-quality prey species. It is therefore evident that the consumption of low-quality food by females has even greater far-reaching effects, detectable from the earlier stages of breeding, including maternal effects and egg production (before hatching).

Overall, our results suggest that individual differences in prey consumption prior to breeding influenced the short-term lipid metabolic profile and reproductive performance of female BFBO. Some studies have shown that the reproductive performance can be influenced by environmental factors and individual physiology during the reproductive and non-reproductive periods [86,87], but there are also other carry-over effects (e.g., non-breeding habitat, previous breeding attempts, age of birds) that need to be considered to have a broader view of what happens with females breeding performance. Oceanographic conditions during both breeding seasons in our study were favorable (considered to be slightly cold; negative equatorial sea surface temperature [SST] anomalies; Climate Prediction Center http://www.nws.noaa.gov), so females should have experienced favorable breeding conditions due to higher prey availability [88], which probably prevented us from observing inter-annual effects.

BFBO females seem to depend on specific prey (enriched in δ^15^N, with greater available energy and lipid content) that allows them to maximize their reproductive effort. However, environmental changes that modify the regional food web trophic structure (e.g., El Niño-Southern Oscillation event or sea surface warming due to climate change) can cause a decrease in the availability of small pelagic fish, which would mean that only females with better individual foraging skills would be able to breed. Adjustments by females in reproductive investment based on their individual body condition suggest that self-maintenance (survival) is prioritized at the expense of reproductive performance; this is consistent with the life-history theory for long-lived organisms such as seabirds.

## Supporting information

S1 Data Excel file with data on blue-footed booby (*Sula nebouxii*) females during courtship and incubation in 2011–2012. (XLSX)

S2 Data Text file describing the data format in S1 Data. (DOCX)

S1 Fig Relationship between isotopic signatures of female blue-footed boobies (during courtship-incubation period) whole blood and potential prey items. Mean (± SD) stable carbon (δ^13^C) and stable nitrogen (δ^15^N) isotope values before the chick-rearing diet (whole blood: subtracting an assumed diet-tissue discrimination factor of 0.24‰ for δ^13^C and 2.25‰ for δ^15^N; Stauss et al. 2012) and Anchovy, Pacific anchoveta, Pacific thread herring, Common halfbeak, Longfin halfbeak, Group 1 (Blue mackerel, Californian anchovy and Mackerel scad). Diet items are from Isla El Rancho, Sinaloa, Mexico.
(TIF)
