# Correction: High quality diet improves lipid metabolic profile and breeding performance in the blue-footed booby, a long-lived seabird

**DOI:** 10.1371/journal.pone.0196318

**Published:** 2018-04-19

**Authors:** Erick González-Medina, José Alfredo Castillo-Guerrero, Sharon Zinah Herzka, Guillermo Fernández

Fig 1 contains a typographical error. The legend “Triglycerydes” on the Y axis should be “Triglycerides.” Please see the corrected figure here.

**Fig 1:**
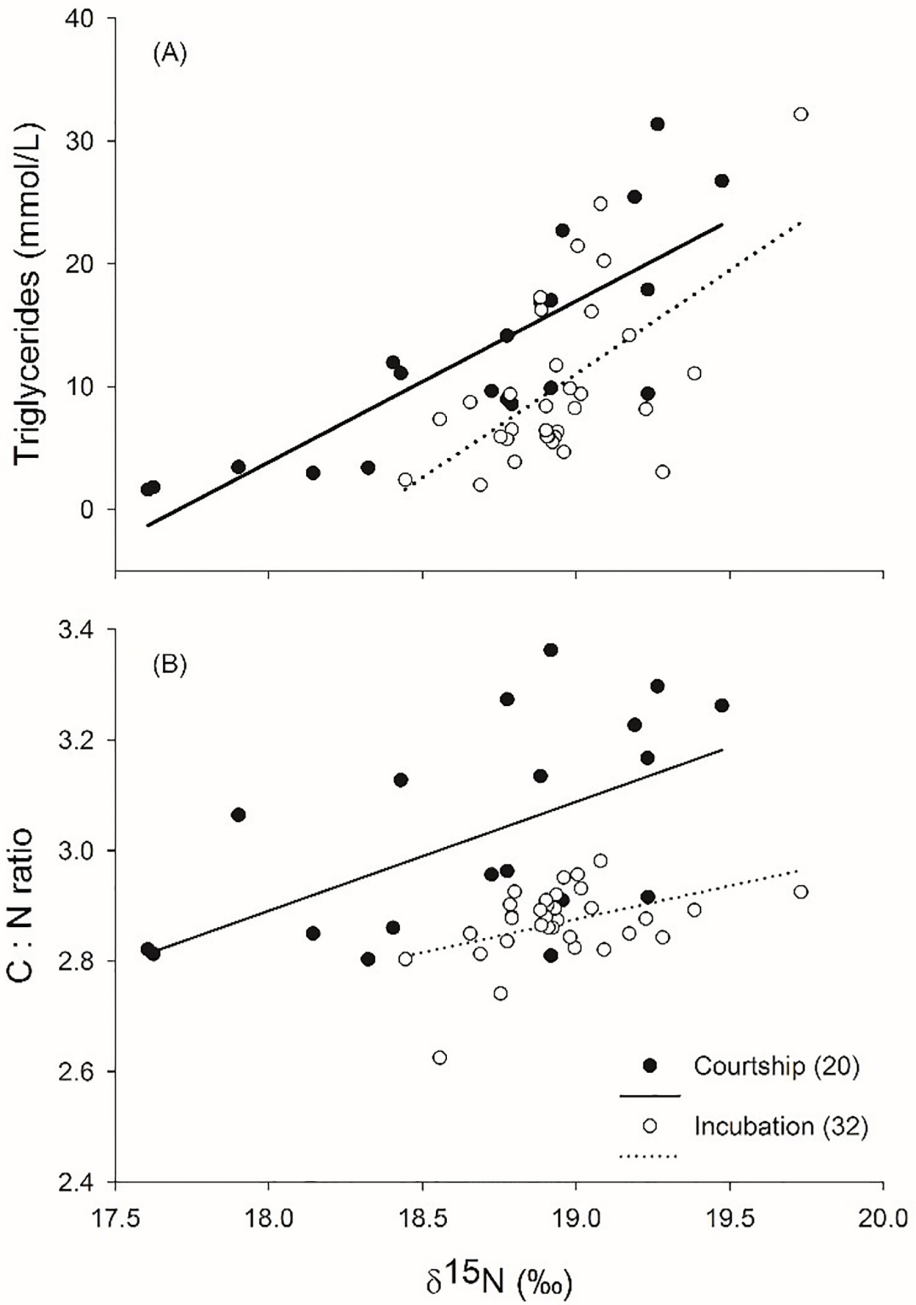
Relationship between δ^15^N values measured in female blue-footed booby (*Sula nebouxii*) whole blood during two reproductive periods (courtship and incubation, sample sizes in parentheses) and (A) triglyceride levels (indicator of body condition) and (B) C:N ratio.

S1 Data contains incorrect data points. Please see the correct dataset here.

## Supporting information

S1 Data Excel file with data on blue-footed booby (*Sula nebouxii*) females during courtship and incubation in 2011–2012. (XLSX)
