# Supplementary material for: High quality diet improves lipid metabolic profile and breeding performance in the blue-footed booby, a long-lived seabird
Source: PLoS One. 2018 Feb 20;13(2):e0193136. doi: 10.1371/journal.pone.0193136 (PMC5819808; doi:10.1371/journal.pone.0193136)
Supplement: S2 Data — (DOCX) [file pone.0193136.s002.docx]

ReadMe for S1_Data.xlsx

The data are in five worksheets:

1) Courtship.

2) Incubation.

3) GLZ.

4) r-Early-late breeders.

5) r-Clutch-size.

**Worksheet 1.** Raw data used to evaluate the relationship between the trophic level (inferred from δ^15^N values) and fasting state (as measured by triglyceride levels) of blue-footed booby (*Sula nebouxii*) females during courtship during the 2012 breeding season, and also to assess whether the isotope composition (δ^15^N) in whole blood of females during the courtship period was related to reproductive parameters (clutch size and laying date).

Each row describes a single value of fasting state (triglyceride levels) and isotope composition (δ^15^N, δ^13^C).

Each observation is recorded as follows: (nest, id, laying date [julian], total egg volume [cm^3^], δ^13^C [‰], C Amount [ug], δ^15^N [‰], N Amount [ug], C:N ratio, triglycerides [mmol/L]).

- “nest” is the number of sampled nest.
- “id” is the alphanumeric code of the ring placed on the bird.
- “laying date (julian)” Is the laying date of the nest converted into julian days.
- “total egg volume (cm^3^)” is the egg volume per clutch calculated by adding the individual egg volumes.
- “δ^13^C (‰)” is the isotopic composition (δ^15^N) in whole blood during the courtship.
- “C Amount (ug)” is the amount of C from the whole blood sample.
- “δ^15^N (‰)” is the isotopic composition (δ^13^C) in whole blood during the courtship.
- “N Amount (ug)” is the amount of N from the whole blood sample.
- “C:N ratio” is a ratio of the mass of carbon to the mass of nitrogen from the whole blood sample.
- “triglycerides (mmol/L)” is the plasma triglyceride levels during the courtship period. The amount of triglyceride was calculated by subtracting the free glycerol from the total triglyceride.

**Worksheet 2.** Raw data used to evaluate the relationship between the trophic level (δ^15^N values) and fasting state (triglyceride levels) of females during incubation by using a general linear model (GLM).

Each row describes a single value of fasting state (triglyceride levels) and isotope composition (δ^15^N, δ^13^C).

Each observation is recorded as follows: (nest, id, year, hatching success [%], δ^13^C [‰],C Amount [ug], δ^15^N [‰], N Amount [ug], C:N ratio, triglycerides [mmol/L]).

- “nest” is the number of sampled nest.
- “id” is the alphanumeric code of the ring placed on the bird.
- “year” is the year when the sample was taken (1 = 2011, 2 = 2012).
- “hatching success (%)” are the eggs hatched / total eggs laid ×100.
- “δ^13^C (‰)” is the isotopic composition (δ^15^N) in whole blood during incubation.
- “C Amount (ug)” is the amount of C from the whole blood sample.
- “δ^15^N (‰)” is the isotopic composition (δ^13^C) in whole blood during incubation.
- “N Amount (ug)” is the amount of N from the whole blood sample.
- “C:N ratio” is a ratio of the mass of carbon to the mass of nitrogen from the whole blood sample.
- “triglycerides (mmol/L)” is the plasma triglyceride levels during incubation period. The amount of triglyceride was calculated by subtracting the free glycerol from the total triglyceride.

**Worksheet 3.** Raw data used for the generalized linear model (GLZ) analysis.

Each row describes a single value of hatching success (success or failure) and isotope composition (δ^15^N).

Each observation is recorded as follows: (nest, id, eggs, hatching success [1,0], laying order, year, δ^15^N [‰]).

- “nest” is the number of sampled nest.
- “id” is the alphanumeric code of the ring placed on the bird.
- “eggs” is the total eggs laid.
- “hatching success (1,0)” Are the categories assigned to the success or failure of hatching of each egg (successful hatching = 1, no hatching = 0).
- “laying order” is the laying order of eggs (a = first egg laid; b = second egg laid; c = third egg laid).
- “year” is the year when the sample was taken (1 = 2011, 2 = 2012).
- “δ^15^N (‰)” is the isotopic composition (δ^13^C) in whole blood during incubation.

**Worksheet 4.** Data source used in SIAR Bayesian multi-source isotopic mixing model in R to estimate the contribution of potential prey to the diets of nesting females (early or late breeders).

Each row describes a single value of nesting females (early or late) and their isotope composition (δ^15^N, δ^13^C).

Each observation is recorded as follows: (code, δ^13^C (‰), δ^15^N (‰)).

- “code” 1 = early breeders; 2 = late breeders.
- “δ^13^C (‰)” is the isotopic composition (δ^15^N) in whole blood during the courtship.
- “δ^15^N (‰)” is the isotopic composition (δ^13^C) in whole blood during the courtship.

**Worksheet 5.** Data source used in SIAR Bayesian multi-source isotopic mixing model in R to estimate the contribution of potential prey to the diets of nesting females as a function of clutch size (0-3 eggs).

Each row describes a single value of clutch size (0-3 eggs) and female’s isotope composition (δ^15^N, δ^13^C).

Each observation is recorded as follows: (code, δ^13^C (‰), δ^15^N (‰)).

- “code” 1 = 0 eggs laid; 2 = one egg laid; 3 = two eggs laid; 4 = three eggs laid.
- “δ^13^C (‰)” is the isotopic composition (δ^15^N) in whole blood during incubation.
- “δ^15^N (‰)” is the isotopic composition (δ^13^C) in whole blood during incubation.
