# Supplementary figures and images for: High quality diet improves lipid metabolic profile and breeding performance in the blue-footed booby, a long-lived seabird
Source: PLoS One. 2018 Feb 20;13(2):e0193136. doi: 10.1371/journal.pone.0193136 (PMC5819808; doi:10.1371/journal.pone.0193136)

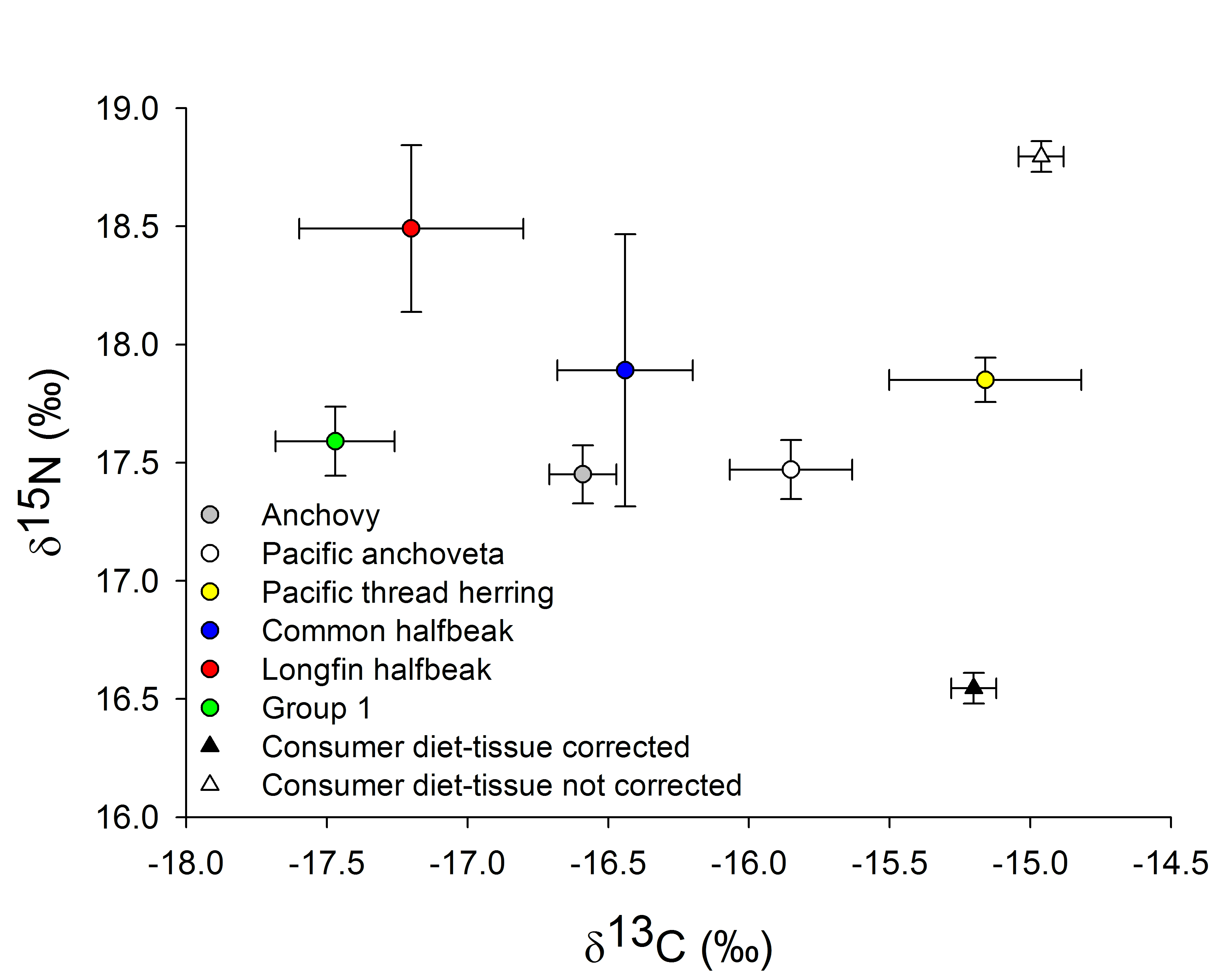

Supplement: S1 Fig — Mean (± SD) stable carbon (δ13C) and stable nitrogen (δ15N) isotope values before the chick-rearing diet (whole blood: subtracting an assumed diet-tissue discrimination factor of 0.24‰ for δ13C and 2.25‰ for δ15N; Stauss et al. 2012) and Anchovy, Pacific anchoveta, Pacific thread herring, Common halfbeak, Longfin halfbeak, Group 1 (Blue mackerel, Californian anchovy and Mackerel scad). Diet items are from Isla El Rancho, Sinaloa, Mexico. (TIF) [file pone.0193136.s003.TIF]
